# Proliferation in the Alzheimer Hippocampus Is due to Microglia, Not Astroglia, and Occurs at Sites of Amyloid Deposition

**DOI:** 10.1155/2014/693851

**Published:** 2014-08-19

**Authors:** Michael W. Marlatt, Jan Bauer, Eleonora Aronica, Elise S. van Haastert, Jeroen J. M. Hoozemans, Marian Joels, Paul J. Lucassen

**Affiliations:** ^1^Swammerdam Institute for Life Sciences-Center for Neuroscience, University of Amsterdam, Science Park 904, 1098 XH Amsterdam, The Netherlands; ^2^Department of Neuroimmunology, Center for Brain Research, Medical University of Vienna, Spitalgasse 4, 1090 Vienna, Austria; ^3^Department of Neuropathology, Academic Medical Center, University of Amsterdam, Meibergdreef 9, 1105 AZ Amsterdam, The Netherlands; ^4^Department of Pathology, VU University Medical Center, P.O. Box 7057, 1007 MB Amsterdam, The Netherlands; ^5^Department of Neuroscience and Pharmacology, University Medical Center Utrecht, P.O. Box 85060, 3508 AB Utrecht, The Netherlands

## Abstract

Microglia and astrocytes contribute to Alzheimer's disease (AD) etiology and may mediate early neuroinflammatory responses. Despite their possible role in disease progression and despite the fact that they can respond to amyloid deposition in model systems, little is known about whether astro- or microglia can undergo proliferation in AD and whether this is related to the clinical symptoms or to local neuropathological changes. Previously, proliferation was found to be increased in glia-rich regions of the presenile hippocampus. Since their phenotype was unknown, we here used two novel triple-immunohistochemical protocols to study proliferation in astro- or microglia in relation to amyloid pathology. We selected different age-matched cohorts to study whether proliferative changes relate to clinical severity or to neuropathological changes. Proliferating cells were found across the hippocampus but never in mature neurons or astrocytes. Almost all proliferating cells were colabeled with Iba1+, indicating that particularly microglia contribute to proliferation in AD. Proliferating Iba1+ cells was specifically seen within the borders of amyloid plaques, indicative of an active involvement in, or response to, plaque accumulation. Thus, consistent with animal studies, proliferation in the AD hippocampus is due to microglia, occurs in close proximity of plaque pathology, and may contribute to the neuroinflammation common in AD.

## 1. Introduction

Alzheimer's disease (AD) is an age-associated chronic neurodegenerative disease and the most common form of dementia. Its two main pathological lesions are amyloid-*β* (A*β*) plaques and neurofibrillary tangles (NFTs) that advance through the brain in a hierarchical manner, with the hippocampus being affected strongly, and early, in the disease [1–3]. In addition to A*β* plaques and NFTs, neuroinflammation and glial changes are prominent during AD [4, 5], and epidemiological evidence, for example, indicates that anti-inflammatory drugs may reduce the risk for AD [6, 7].

Neuroinflammation is prominent during AD [8–10] and microglia have consistently been implicated in brain aging, neuroinflammation, and neurodegeneration [11–13]. Multiple roles have been established for these immune cells during health and disease [14–17]. Activated microglia are morphologically identifiable by their expression of Iba1, an inducible protein that regulates actin remodeling [18] and typically have an amoeboid or rod-like morphology, as opposed to minimally activated microglia cells, that have short, ramified processes. Microglia are broadly implicated in AD [4, 8, 19] and have, for example, been found to migrate to A*β* lesions [20–23], degrade A*β* peptide* in vitro* [24, 25], and participate in the clearance of A*β* from the brain [26].

Earlier work had established that microglia can also respond to damage by undergoing proliferation. Indeed, cultured microglia become activated by A*β*; their proliferation index increases and they release tumor necrosis factor *α* (TNF*α*) in response to A*β* [27]. Microgliosis also occurs in AD models [28, 29] and microglial cells undergo proliferation in a transgenic AD mouse model, where markers for proliferation were coexpressed parallel to the development of neuropathological hallmarks, with proliferating microglia seen in the periphery of A*β* plaques [30, 31].

Another common feature of AD is extensive astrogliosis. Astrocytes have been classically known to change in AD and show, for example, hypertrophy or changes in the expression of specific GFAP isoforms, parallel to the development of A*β* plaque pathology [31, 32]. Amyloid plaques can, for example, show intense astrocytic participation [33–37]. Also, activation and recruitment of microglia may occur in concert with astrocytes; microglia secrete interleukin-1 (IL-1), a major cytokine, and potent activator of astrogliosis [38, 39]. While astrocyte proliferation has been seen in response to, for example, stab wounds [40], their role in the neuropathology of AD remains elusive, and it is, for example, unknown if astrocytes proliferate in AD or in response to A*β* lesions in human brain.

We previously documented an increase in proliferation in the hippocampus of presenile AD patients (≤70 years of age) [41]. Interestingly, these increases were particularly present in glia-rich regions and not in the main neuronal layers, for example. Given this location and the roles of microglia and astroglia in disease, we anticipated astro- and/or microglia might contribute to the proliferation seen in the AD hippocampus. As the phenotype of these proliferating cells and their relationship to A*β* plaques were unknown, we therefore developed two triple-immunohistochemical stainings to detect astrocyte or microglial proliferation in combination with A*β* pathology. To further assess whether the clinical or neuropathological aspects of dementia influence proliferation, we here studied older age-matched individuals that differed in cognitive and neuropathological staging: (1) a control (Con) cohort without any clinical or neuropathological signs of dementia, (2) a cohort that had low levels of AD pathology at autopsy (Braak stages 1-2) but was clinically demented during their lives (Dem), and (3) a confirmed Alzheimer cohort (AD) that was clinically demented and exhibited severe neuropathology at autopsy (Braak stages 4-5). As followup to our study on presenile cases, we here asked (a) whether also elderly cohorts (>80 years) of AD or demented subjects exhibit increases in proliferation, (b) if astrocytes (GFAP+) or microglia (Iba1+) contribute to these proliferative changes, and (c) if proliferation is anatomically related to A*β* pathology or to clinical severity.

## 2. Materials and Methods

### 2.1. Subjects

Hippocampal brain tissue was obtained via The Netherlands Brain Bank rapid autopsy program in accordance with all local ethical legislation and in accordance with the ethical standards laid down in the 1964 Declaration of Helsinki. Signed informed consent was available for all patients in the study. Generally after short postmortem delays, hippocampal tissue was dissected and then fixed in 10% buffered formalin for 1-2 months and dehydrated before paraffin embedding. 8 *μ*m sections were mounted on Superfrost Plus slides. Hippocampal tissue was selected from 8 age-matched, nondemented controls (Con), 8 clinically demented (Dem) cases with low Braak scores, and 8 confirmed Alzheimer disease (AD) cases. Braak staging of the three groups was, respectively, Con: 1.4 ± 0.2, Dem: 1.5 ± 0.2, and AD: 4.8 ± 0.3. Mean age (y) was, respectively, Con: 80 ± 3 y, Dem: 84 ± 2, and AD: 81 ± 2. Brain weight (g) was Con: 1208 ± 65, Dem: 1238 ± 87, and AD: 1125 ± 50. No significant differences in postmortem delay (between 5–7 hours) or fixation duration (around 1 month in formalin) were present and the groups were further balanced male : female in a 1 : 1 ratio (Table 1).

### 2.2. Immunohistochemistry

Sections were deparaffinized in xylene and rehydrated through graded ethanol solutions and then washed in 0.05 M PBS prior to antigen retrieval by heating in a food steamer (MultiGourmet FS 20, Braun, Kronberg/Taunus, Germany) for 60 minutes at 100°C in citrate buffer (pH = 6.0). Sections were cooled to room temperature prior to incubation with 0.3% H_2_O_2_ to quench endogenous peroxidase activity. Primary antibodies were diluted in Dako Washing Buffer (Dako S3006, Glostrup, Denmark) supplemented with 10% fetal calf serum and incubated for 1 hr at room temperature (RT) and then overnight at 4°C.

Triple immunostaining was performed through sequential development steps. First, anti-A*β* antibody (Ab) (1 : 2,000 MAB1561, clone 4G8, Millipore, Billerica, MA, USA) was reacted with biotinylated sheep anti-mouse (1 : 500 Jackson ImmunoResearch, West Grove, PA, USA), followed by incubation with avidin-peroxidase (Sigma, Germany) and development with diaminobenzidine (DAB, Sigma, Germany) substrate. After washing, sections were incubated with antiproliferating cell nuclear antibody (PCNA) Ab (1 : 25,000 Dako M0879, Glostrup, Denmark) overnight, followed by donkey anti-mouse alkaline phosphatase-conjugated Ab and NBT/BCIP substrate to produce a blue stain. In order to inactivate the binding properties of the first round of antibodies and to retrieve additional GFAP and Iba1 epitopes, sections were then treated with EDTA (10 mM, pH = 9.0) in TRIS buffer for 30 minutes followed by washing and final incubation with rabbit polyclonal anti-GFAP (1 : 1,500 Dako Z0334, Glostrup, Denmark) or anti-Iba1 Ab (1 : 1,500 kindly provided by Dr. S. Kohsaka, National Institute of Neuroscience, Tokyo, Japan). Subsequently, sections were incubated with alkaline phosphatase-conjugated donkey anti-rabbit (Jackson ImmunoResearch, USA) and developed with Fast Red substrate to stain, in separate series, astrocytes and microglia dark red.

All secondary antibodies were incubated for 1 hr at room temperature. The anti-A*β* 4G8 antibody was found previously to provide the most consistent staining of diffuse plaques in formalin-fixed, paraffin embedded human tissue [42]. 4G8 Ab (recognizing amino acids 17–24 of A*β* peptide) recognizes extracellular APP domains containing the A*β* epitope and thus labels full length APP as well as A*β* plaques. PCNA has been validated and used before extensively and the anti-PCNA antibody identifies proliferating cell profiles in human brain processed in a similar manner [43–54].

Glial fibrillary acidic protein (GFAP) is a classic member of the intermediate filament protein family involved in astrocyte cytoarchitecture and generally considered a sensitive marker of reactive astrogliosis in the CNS [55, 56]. The pan-GFAP antibody we have used identifies a 50 kDa intracytoplasmic fibrillary acidic protein that is constitutively and specifically expressed in the cytoskeleton of all astrocytes, including differentially spliced isoforms [56]. Activation of astrocytes, that is, reactive gliosis, is generally reflected by increased expression of GFAP per cell and an upregulation of the intermediate filament network, often paralleled by morphological changes.

For the detection of microglia, several markers, like Iba1 and CD68 antibodies [57], are available that identify specific activational stages and/or types of microglia [58, 59]. We here selected the Iba1 antibody, a 17 kDa ionizing calcium-binding adaptor molecule that is specifically expressed in all macrophages and microglia and is upregulated during their activation in human brain. Iba1 has further been validated before and shown to identify general activation of microglia [60] in various CNS disorders [58] like Creutzfeldt-Jakob disease [61], brain tumor [62], and influenza encephalitis [63, 64].

### 2.3. Morphometry and Quantification

Cross-sectional areas for the dentate gyrus and CA subregions were determined using StereoInvestigator software (MicroBrightField Inc., USA) linked to a Zeiss Axiophot microscope (Carl Zeiss AG, Germany) to outline the appropriate hippocampal subregions. All phenotypic quantification was performed in midlevel sections of the hippocampus. Cell count values were normalized to the surface area of each anatomical subregion and expressed per mm^2^. Quantification of PCNA+ and Iba1+ cells was obtained from the PCNA/Iba1/A*β* stained slides, while quantification of GFAP+ cells was obtained from the PCNA/GFAP/A*β* triple stained sections. Values for cell numbers are expressed as averages ± SE and values for plaques are expressed as plaques per mm^2^.

## 3. Results

### 3.1. Proliferation Occurs across Disease Cohorts and Hippocampal Subregions

**Figure 1:**
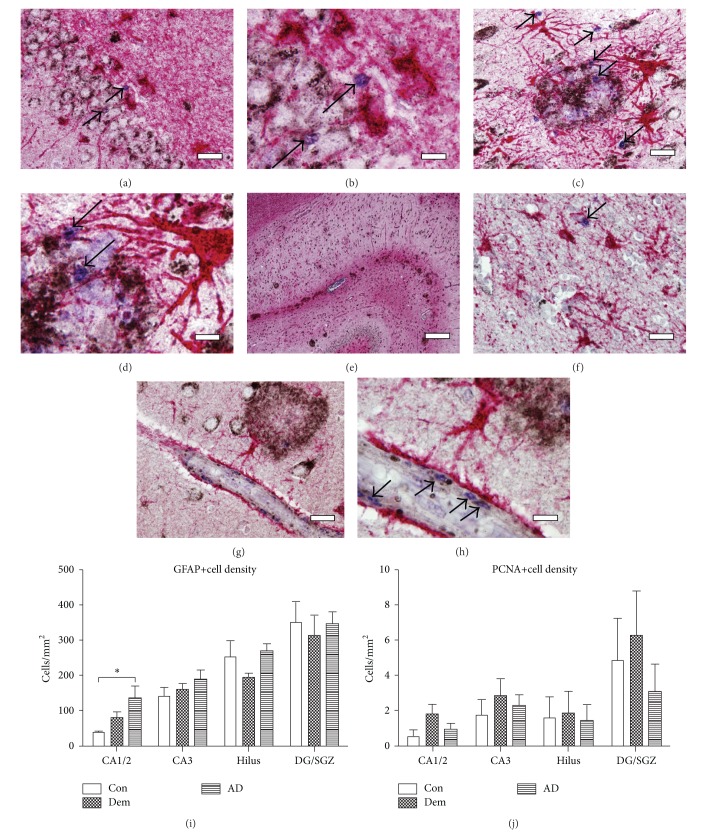
GFAP and PCNA expression in the human hippocampus (a, b) Immunolabeled GFAP+ (red) and PCNA+ (blue) cells did not colocalize in the SGZ or other areas of the hippocampus. (c-d) PCNA+ cells (arrows) were observed in the presence of hypertrophic astrocytes with processes infiltrating A*β* plaques (black). (e) A low-magnification image representative of GFAP and A*β* plaques distributed through out the CA areas, with heavy immunostaining in the stratum moleculare and stratum lacunosum. (f) PCNA+ cells were also found in glia-rich regions in the absence of A*β* plaques and in blood vessels (g, h) outlined by astrocytic endfeet. (i) GFAP+ cells were found at higher density in the CA1/2 region in the AD versus control subjects (one-way ANOVA *P* < 0.001). (j) PCNA+ cell density was not significantly different between patient cohorts. Scale bars: left panels (a, c, g) = 50 *μ*m, panel (e) = 500 *μ*m. Right panels (b, d, h) = 20 *μ*m, panel (f) = 50 *μ*m.

We first studied GFAP+ and PCNA+ cells across cohorts to evaluate any association between the two protein markers. We found PCNA+ cells at a low density throughout the main subregions of the hippocampus, including the granule cell layer (GCL) and subgranular zone (SGZ) of the dentate gyrus (DG) (Figures 1(a) and 1(b)), areas populated with granule neurons and neural stem cells, respectively. PCNA was never seen within GFAP+ astrocytes or in granule neurons labeled by 4G8 immunoreactivity with membrane-bound APP. PCNA+ cells were however seen adjacent to GFAP+ cells in the hilus and cornus ammonis (CA) regions of the hippocampus. These cells were typically small and did not display membrane-bound APP as observed in adjacent granule and pyramidal neurons. Hypertrophic GFAP+ astrocytes that were seen to infiltrate A*β* plaques were not colabeled with PCNA in any hippocampal subregion (Figures 1(c) and 1(d)). Regardless of astrocyte morphology, location, or plaque association, GFAP+ cells were never found to be colabeled with PCNA.

Within the CA regions, PCNA+ cells were also found in astrocyte-rich regions that lacked A*β* pathology (Figure 1(f)), and, consistent with our previous findings, they were also present in the vascular epithelium (Figures 1(g) and 1(h)). The population of PCNA+ cells was not significantly elevated in the current, older AD cohort. In contrast, the DEM cohort showed a trend for high proliferation compared to the other groups (Figure 1(j)). Quantification of PCNA+ cells showed a clear increase in proliferation; however, this was not significant: Con: 3 ± 1 cells, Dem: 18 ± 5 cells, and AD: 12 ± 3 cells within the CA1/2 subregion (one-way ANOVA *P* = 0.07).

GFAP+ astrocytes showed a characteristic, spider-like cellular morphology. They were seen in close proximity to neurons and blood vessels with endfeet clearly visible that contacted blood vessel walls. In the CA regions and subiculum, hypertrophic astrocytes were observed with processes infiltrating diffuse, primitive, and dense-core A*β* plaques (Figures 1(c) and 1(d)). These cells, when compared to astrocytes not associated with plaques, frequently showed upregulation of the intermediate filament network, a sign of reactive gliosis, although not all astrocytes close to plaques (within 50 *μ*m) showed this hypertrophic morphology. GFAP expression was enriched in the stratum moleculare and stratum lacunosum of the AD hippocampus, where astrocytes were found preferentially with A*β* plaques (Figure 1(e)). A significantly increased population of GFAP+ cells was observed in the CA1/2 area in the AD patient cohort compared to controls (Figure 1(i)) (one-way ANOVA *P* = 0.02).

Plaque values were low across the Braak 1 stage cases, the control cohort, and dementia cohort. Morphologically, the plaque pathology in the AD cohort was distinct from the control and dementia sections; AD cases had a significant increase in dense-core (one-way ANOVA *P* < 0.01) and GFAP-associated, that is, degraded plaque subtypes (one-way ANOVA *P* < 0.001). For the dense-core plaques, averaged numbers were as follows: control: 0.25+/−0.2, dementia: 0.25+/−0.2, and AD: 2.37+/−0.8. For the degraded plaques, these numbers were control: 0.37+/−0.2, dementia: 1.37+/−1.1, and AD: 7.25+/−1.89. The population of A*β* plaques with invasive hypertrophic GFAP+ astrocytes was increased in the AD population (one-way ANOVA *P* < 0.001). However, we could not observe any indication that mature astrocytes underwent proliferation in the presence of A*β* plaques.

### 3.2. Microglia Proliferate in All Hippocampal Subregions but Their Morphology Is Unchanged across Conditions

**Figure 2:**
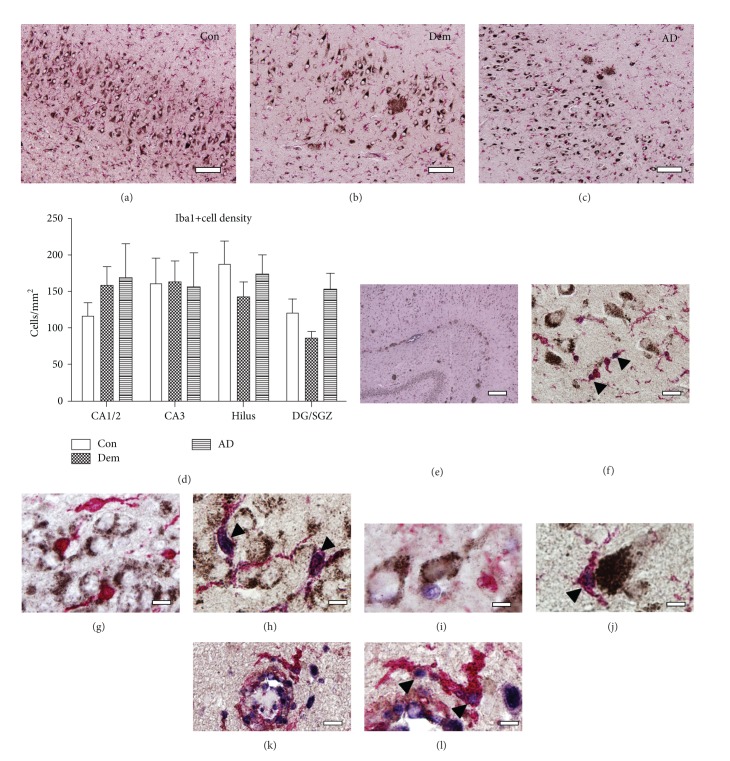
Iba1 morphology and proliferation in human hippocampus. (a–c) Representative images for Con, Dem, and AD subjects, respectively, illustrating a similar morphology of Iba1+ (red) cells. (d) Quantitative analysis of Iba+ cell density showed no significant difference between cohorts. Ramified Iba1+ cells throughout the hippocampus coexpress PCNA (arrowheads): (e) representative A*β* and Iba1 distribution, (f) ramified and proliferating Iba1+ cells in the CA3 in the absence of A*β* plaques, (g-h) in the GCL across patient cohorts, and (i-j) near pyramidal neurons expressing APP (black). (k-l) Positive control section from a brain infected with herpes simplex virus encephalitis (HSVE). Infected vessels show activated Iba1+ microglia and coexpression of PCNA (arrowheads). Scale bars (a–c) = 100 *μ*m, (e) = 500 *μ*m, (f) = 50 *μ*m, (g–j) = 20 *μ*m, (k) = 50 *μ*m, and (l) = 20 *μ*m.

Since GFAP+ astrocytes were not colabeled with PCNA, we next used combined Iba1-PCNA double immunocytochemistry to test whether proliferation did occur in microglial cells. Iba1+ microglia had a ramified morphology with observable processes but there were neither morphological alterations nor quantitative changes in Iba1 expression across the 3 cohorts (one-way ANOVA *P* = 0.43) (Figures 2(a)–2(d)). Furthermore, local expression of Iba1 per se was not associated with areas enriched for GFAP expression or A*β* deposits, like the stratum moleculare and stratum lacunosum (Figure 2(e)).

Microglia within the hippocampal CA subregions are colabeled with PCNA+ (Figure 2(f)), while Iba1+/PCNA+ coexpression was also seen in the DG and SGZ (Figures 2(g) and 2(h)). We closely inspected mature pyramidal neurons for evidence of PCNA expression but never observed neurons colabeled with PCNA, although clear examples of proliferating and nonproliferating microglia were found adjacent to mature pyramidal neurons (Figures 2(i) and 2(j)). Tissue sections from a herpes simplex virus (HSV) encephalitis brain sample were included as positive controls (Figures 2(k) and 2(l)) since HSV encephalitis is observed as an acute focal, necrotizing inflammation, and infection of neurons is thought to occur after infection of vascular endothelium [65]. In agreement, numerous PCNA+/Iba1+ cells were found in blood vessels in these cases. The Iba1+ cells in these positive control samples had an amoeboid morphology, unlike and clearly different from the ramified cells found in our aged patient cohort, indicating active clearance of cellular debris. Average numbers of Iba1+ cells per group were as follows: control: 116.54+/−18.1, dementia: 158.43+/−26.0, and AD: 170.79+/−42.2.

### 3.3. A*β* Plaque Load, Morphology, and Degradation

**Figure 3:**
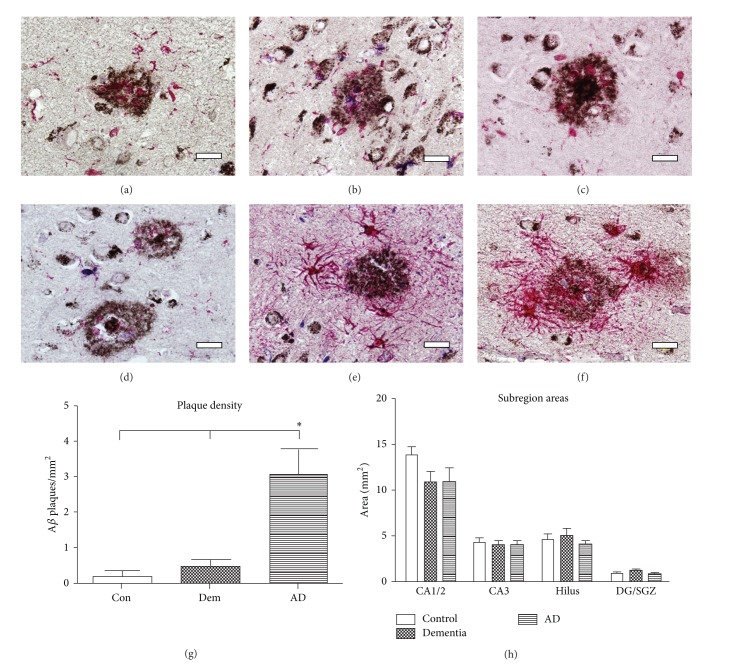
Glia are tightly associated with A*β* plaque morphology. The same chromogen Fast Red substrate was used to visualize either microglia (a–d) or astrocytes (e–h) in these images; (a–d) Iba1+ cells infiltrate A*β* plaques and appear involved in the formation of dense-core plaques: (b) proliferating Iba1+ cells (arrowhead) are seen within primitive plaques, (c) a dense-core plaque with concentric Iba1+ cells, and (d) a mature dense-core plaque without concentric Iba1+ cells. (e-f) GFAP+ astrocytes participate in degradation of multiple plaque subtypes; proliferating cells (arrowheads) within the borders of plaques degraded by GFAP+ astrocytes. Scale bars (a–f) = 50 *μ*m. (g) Plaque load was significantly increased in the AD cohort compared to Con and Dem cohorts (one-way ANOVA *P* < 0.001). (h) Hippocampal cross-sectional areas were not significantly different.

Similar to earlier studies, the 4G8 antibody labeled diffuse, primitive, dense-core, and remnant A*β* plaque profiles [33]. Remnant plaques showed intense astrocytic participation with ragged edges indicative of degradation. The control and dementia groups had a similar degree of A*β* staining in the hippocampus; A*β* plaques in control and dementia cases were mainly confined to the subiculum and parahippocampal gyrus. As expected based on the Braak scores, total A*β* plaque load was significantly increased in the hippocampus of the AD cases compared to the control and dementia groups (average of 3 plaques in the AD versus 0.10/mm^2^ in Con group) (one-way ANOVA *P* < 0.001) (Figure 3(g)). Morphologically, the plaque pathology in the AD cohort was distinct from the control and dementia sections; AD cases had a significant increase in dense-core (one-way ANOVA *P* < 0.01) and GFAP-associated, that is, degraded plaque subtypes (one-way ANOVA *P* < 0.001).

Interestingly, Iba1+ cells often formed a concentric ring around A*β* dense-cored plaques and were generally found in close association to plaques (Figures 3(a)–3(d)). Iba1+/PCNA+ double-labeled cells were visible within the borders of A*β* plaques (Figure 3(b)). Also, PCNA+ single-labeled cells were found associated with degraded plaques, often within 200 *μ*m of all plaque subtypes (Figures 3(e) and 3(f)). The majority of plaques, however, did not show evidence of proliferating PCNA+/Iba1+ microglia.

Despite this evidence that microglia proliferate directly at sites of A*β* deposition and in proximity to plaque-laden areas, there was no quantitative increase in the overall Iba1+ cell numbers in the AD compared to the DEM and CON cohorts. Similarly, no statistically significant increases were found in the number of Iba1+ cells that colocalized with A*β* plaques in AD compared to the control and dementia cohorts (one-way ANOVA *P* = 0.49).

After the surface areas of the hippocampal subregions were measured, no significant differences were observed across subregions; however, surface areas of the CA1/2 subregion were somewhat smaller, but not significantly, in the AD cohort (one-way ANOVA *P* = 0.14, Figure 3(h)).

## 4. Discussion

We investigated proliferation in the human hippocampus of control subjects, of clinically demented cases with low Braak scores, and in clinically as well as neuropathologically confirmed AD patients. We focused on PCNA+ proliferating cells, their colocalization with astro- or microglia, and their location relative to A*β* plaque pathology and questioned whether cognitive status or the neuropathology modulated these readouts. Proliferating cells were found throughout the hippocampal subregions, including the CA areas and astrocyte-rich regions. In contrast to younger, presenile cases we had studied before [41], proliferation in the present older cohorts, demented or AD, was not different from age-matched controls. PCNA+ profiles were never observed in mature neurons or GFAP+ astrocytes, indicating that these cell types do not proliferate or reenter the cell cycle during dementia or AD. Iba1+ microglia, however, did coexpress PCNA across cohorts. These cells were specifically present within the borders of A*β* plaques, indicating that microglia proliferate actively and directly at the site of A*β* deposition. Although amyloid pathology is much more complex than plaques alone [66], our observations agree with previous findings on proliferation in glia and on microglial participation in the formation and maintenance of A*β* plaques in AD [21–23, 52].

In contrast to our previous study on younger AD patients (mean age 66 years) [41], we here studied 3 older cohorts with mean ages of 81 years and found no differences in proliferation between the groups, indicating that brain plasticity is further reduced in subjects this old. Also, we show that particularly the microglia and not astroglia cells are responsible for the proliferation in the hippocampus in these conditions. In our current study, although only a trend was present, proliferation was highest in CA1/2, CA3 and DG of the dementia cohort relative to both the controls and AD cases. This suggests that if proliferation of microglia cells impacts cognition, this apparently occurs independent of the Braak staging that was very low in the Dem group. The morphology of the microglial cells further indicated that these cells, whether or not they colabeled for PCNA, were minimally activated, particularly when compared to the morphology of microglia in Parkinson's disease patients [57, 67, 68] or to the hyper-ramified and rod-like Iba1+ morphologies observed, for example, in pediatric epilepsy [69]. Our present Iba1 stainings highlight that microglia do not show dramatic changes in quantity or morphology during AD, particularly when compared to other severe neurological disorders, but that a subset expresses proliferation markers.

Whereas, in general, aged microglia do not show major morphological changes, there is evidence that these cells, in functional terms, are highly proinflammatory.* Ex vivo* microglia cultures isolated from aged mice, for example, show elevated production of the proinflammatory molecules Il-6 and TNF-*α*, while microglia from old animals have a decreased ability to internalize A*β* compared to cells isolated from young animals [70]. Microglial and astroglial responses may further be coordinated in a concerted manner that increases neuroinflammation [5]. For instance, increased levels of glia maturation factor (GMF) were reported in the periphery of A*β* plaques [71]. Interestingly, GMF expressed in astrocytes enhances production of TNF-*α*, Il-1*β*, Il-6, and IP-10 by microglia [72]. Hence, a combination of primary astrocytic and microglial responses may occur during plaque deposition. As each cell type likely responds differentially to the development and presence of A*β* plaques, microglial proliferation and astrocytic activation may be complementary processes that could enhance local inflammation at A*β* plaque sites. Activation of astrocytes is generally reflected by increased expression of GFAP per cell or proteasome activation [17, 31] and hardly ever by proliferation. It will be of interest to address the relationship between and consequences of these two events in future studies.

While proliferating microglia are seen in the hippocampus, the source of these cells remains to be determined. CNS microglia are unique because two populations exist, that is, resident cells present since early development and infiltrating microglia that pass the blood-brain barrier (BBB). Iba1 identifies both types of microglia in the brain regardless of their source. Determining the kinetics of microglial infiltration during a chronic disease like AD is challenging, but so far resident microglia were shown to incorporate the thymidine analog BrdU+ in AD mice [30] and during the early stages of experimental autoimmune encephalitis [49]. Resident cell self-renewal, but not infiltration, was found to be responsible for local microglia expansion in an earlier study [73]. In AD, circulating cells may be involved as well;* in vitro* studies have shown that the recruitment of monocytes across the BBB is increased after an interaction of the A*β* peptide with RAGE receptor [74]. In AD mouse models, bone marrow-derived microglia can drastically reduce plaque burden [75]. Hence, the source and role of each specific population, that is, resident versus infiltrating, is challenging to study in end-stage human brain tissue and thus remains to be determined during different stages of AD and in AD mouse models.

Another relevant issue in this respect is the turnover of the microglia population. Although limited information is available on their precise kinetics, glial cells exhibit remarkable homeostasis and strive to maintain a constant density in response to internal or external perturbations. In a recent paper by Elmore et al. [76], selective but global depletion of the microglia population understanding how this process of cellular maintenance is regulated and identifying the progenitors responsible for replenishing distinct classes of glia could lead to new strategies for speeding recovery from injury.

Our results on proliferation in brain microvessels are in agreement with earlier studies in which proliferating cells were found associated with the vasculature [41, 77]. These cells may represent perivascular macrophages or monocytes becoming perivascular macrophages [77]. Alternatively, PCNA+ expression associated with the vasculature may correspond to damage to blood vessels. Stroke-associated conditions and oxidative endothelial injury can induce PCNA expression in vascular smooth muscle cells isolated from brain arterioles [78, 79]. From the records of our patients, however, there are no indications that this has played a significant role.

After careful study, PCNA+ profiles were never present in mature hippocampal neurons. Earlier studies using various cell cycle markers on human AD or Lewy body dementia tissues suggested that a subset of pyramidal neurons in the affected human hippocampus may reenter the cell cycle during early stages [54, 80–82]. Other cell-cycle makers, including Ki-67, cyclin B1, cyclin D, and cdk4, have been observed in mature neurons during prodromal stages of AD [80–84]. These neurons were often also immunoreactive for intraneuronal accumulations of paired helical filament tau [84]. Also, expression of PCNA in mature hippocampal neurons has been seen during a neuronal death cascade [81–83, 85] while other reports showing increased neuronal aneuploidy further suggest that cell cycle protein expression occurs mainly during early AD [52, 54, 81, 82, 85]. Our study does not conflict with this literature as we first included mainly old, end-stage cases. Second, our triple-staining protocol was specifically developed to identify proliferation in glia and a number of methodological differences exist between our protocol and those identifying neuronal cell cycle reentry. We employed a unique antigen retrieval technique with a low concentration of PCNA antibody to create a sensitive method for detecting proliferating glia and perivascular macrophages, consistent with previous neuropathological studies [86].

Our study further agrees with recent findings from the APPswePS1dE9 AD mouse model. It has been established that CD11b microglia cell clusters were observed as early as 4 months of age [87]. Other studies established that particularly the microglia cells proliferate around A*β* plaques, without astrocyte proliferation in these animals; indeed, age-related increases in proliferation in this mouse model were due to increases in newborn BrdU+ cells coexpressing markers for activated microglia [30]. Despite its limitations, postmortem histological investigation of human brain remains important, particularly since modeling the participation of glia to neurodegenerative diseases in transgenic mice has so far provided conflicting results. For instance, microglia played almost no role in the formation and maintenance of A*β* plaques in transgenic mice that lacked microglia [88] whereas others have shown microglial engagement in dense-cored plaques [89] with the capacity to reduce plaque burden [75, 90–92]. However, transgenic mice seldom recapitulate the regional variability of A*β* plaques seen in human AD, suggesting that inbred mouse models are not always optimal for studying the complex* in situ* microglial responses in human brain [93].

In conclusion, we demonstrate that proliferation in the human hippocampus is due to microglia and not to that of astroglia or mature neurons. These results are consistent with recent observations in AD mouse models. Although our data do not exclude that stimulation of microglia can also occur through additional mechanisms, they further suggest that microglial proliferation is not influenced by cognitive decline but occurs in close association with, and possibly in response to, local A*β* plaque pathology.

## Figures and Tables

**Table 1:** Summarized patient data for the 3 cohorts of human brain tissue.

Status	ID	Sex	Age	Brain weight (g)	Braak stage
ND	00-137	f	92	1031	1
ND	96-044	f	90	1101	2
ND	98-056	f	83	1000	1
ND	99-052	f	79	1325	2
ND	01-016	m	77	1138	1
ND	01-017	m	79	1334	1
ND	02-087	m	71	1190	1
ND	97-043	m	68	1547	2

Dementia	01-131	f	82	1094	1
Dementia	05-058	f	76	1055	2
Dementia	97-004	f	88	966	2
Dementia	97-047	f	91	1114	1
Dementia	01-075	m	72	1514	1
Dementia	04-022	m	84	1154	2
Dementia	05-004	m	91	1347	1
Dementia	94-090	m	86	1663	2

AD	01-010	f	84	1023	4
AD	03-090	f	87	929	5
AD	05-070	f	77	1178	5
AD	99-095	f	80	1200	6
AD	06-013	m	81	1253	4
AD	06-016	m	72	948	6
AD	93-034	m	78	1315	4
AD	94-121	m	85	1155	4

Hippocampal tissue was obtained from 8 nondemented controls (ND), 8 demented cases with low Braak scores that had suffered from clinical dementia (Dem), and 8 clinically and neuropathologically confirmed senile Alzheimer's disease cases (AD); Braak staging (ND: Braak 1.4 ± 0.2, Dem: 1.5 ± 0.2, and AD: 4.8 ± 0.3). Cases were matched for age (years) (ND: 80 ± 3, Dem: 84 ± 2, AD: 81 ± 2), postmortem delay, fixation duration, and sex.

## Abbreviations

Ab: Antibody
A*β*: Amyloid-*β*
AD: Alzheimer's disease
BBB: Blood-brain barrier
CNS: Central nervous system
Con: Control
CA: Cornus ammonis
Dem: Dementia
DG: Dentate gyrus
GFAP: Glial fibrillary acidic protein
GFAP+: GFAP immunopositive
GMF: Glia maturation factor
HSV: Herpes simplex virus
Iba1+: Iba1 immunopositive
IL-1: Interleukin-1
NFT: Neurofibrillary tangles
P2X_7_R: P2X_7_ receptor
PHF-1: Paired helical filament
PCNA: Proliferating cell nuclear antigen
PCNA+: PCNA immunopositive
RAGE receptor: Receptor of advanced glycation end-products
SGZ: Subgranular zone.
